# The Importance of Project Description to Charitable Crowdfunding Success: The Mediating Role of Forwarding Times

**DOI:** 10.3389/fpsyg.2022.845198

**Published:** 2022-04-27

**Authors:** Liangdong Lu, Weijian Jiang, Jia Xu, Fei Wang

**Affiliations:** ^1^Business School, Hohai University, Nanjing, China; ^2^International School of Business and Finance, Sun Yat-sen University, Guangzhou, China

**Keywords:** charitable crowdfunding, content analysis, crisis involvement, framing effect, information processing

## Abstract

The COVID-19 outbreak has been a public health crisis of international concern, causing huge impact on people’s lives. As an important part of social public crisis management, how to quickly and effectively raise resources to participate in emergency relief in the era of self-media is a common challenge faced by global charitable organizations. This article attempts to use empirical evidence from Tencent charitable crowdfunding platform, the largest charitable crowdfunding platform in China, to answer this question. We consider 205 COVID-19 charitable projects and 11,177,249 donors to assess the process by which non-profit organizations raise funds through the information about project descriptions. Based on the effects of information and emotional framing, we explore the effects of the readability (i.e., complexity and understandability) and negative tone of the project description on fundraising amount. We then investigate the mediating role of forwarding times, as affective response to the text might explain forwarding times, which in turn affects money raised by increasing the visibility of the campaign. On this basis, the moderating role of recipient’s crisis involvement is tested during this process. The empirical results indicate that the complexity of the description will reduce the fundraising amount, while understandability and negative tone help to improve it. Furthermore, we found that forwarding times played an important mediating role in this process. Then the buffer effect of crisis involvement on the negative effect of complexity was validated, and its amplification on the positive effects of understandability was also verified.

## Introduction

**FIGURE 1:**
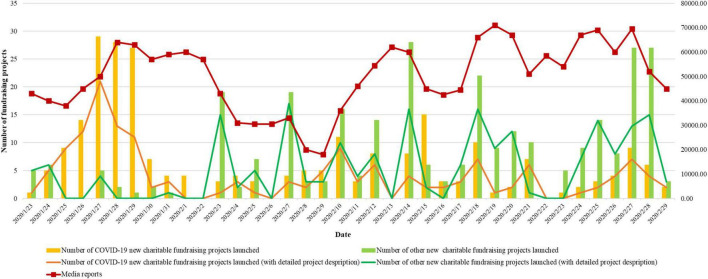
Statistical information of charitable fundraising projects on Tencent and Media reports on web and social media.

Since the outbreak of the novel coronavirus disease (COVID-19), charitable organizations, an important part of social crisis management, have launched a variety of fundraising projects to rapidly raise resources for crisis relief. These projects are primarily transmitted to the public through social media, thereby obtaining financial assistance from a distributed online audience (Gerber and Hui, 2013; Li et al., 2018). Information from Tencent^1^, the largest charitable crowdfunding platform in China, shows that since the Hubei Red Cross launched the first COVID-19 fundraising project on January 23, 2020, almost the entire platform has been dedicated to COVID-19 charity projects within 10 days of the outbreak of the pandemic (see Figure 1). The sponsors of these fundraising projects are nearly 200 charitable organizations throughout China. Hence, how to quickly and effectively raise resources to participate in crisis relief through the charitable crowdfunding platform (hereafter referred to as Tencent) has been a common challenge faced by these charitable organizations. This study uses empirical evidence from Tencent, based on the framing effect, to determine how the content of the project description and the donor’s forwarding times, moderated by the crisis involvement, affects the amount of the fundraising effort.

Before initiating a project on Tencent, initiators must register and provide valid information about their qualifications. The application is reviewed by Tencent and, if approved, an independent project page is established, which includes the project title; a description of the project and photos, the initiator, and executor; an authenticity label; and project metrics, such as status, target, raised money, and deadline. In this model, the basic problem faced by both fundraisers and donors is information asymmetry. The information to support the donor’s decision-making process mainly comes from the project description on the page; therefore, the description provides a forum for fundraisers to persuade potential donors to contribute. Hence, it is crucial to improve the effectiveness of fundraising messages through information framing (Das et al., 2008).

Previous research on financial crowdfunding shows that project attributes, such as the target, duration, and the number of Facebook friends of the initiator, affect the success of the project (Greenberg et al., 2013; Li and Duan, 2014; Mollick, 2014; Xu et al., 2014; Kuppuswamy and Bayus, 2015). Meanwhile, in the humanitarian communication side, based on Text-analytical approaches scholars explored the role of mission statement, donation appeals and humanitarian advertising for charitable organizations. The findings revealed the important effect of textual strategies. Based on this, we will focus on the text description of charity crowdfunding in crisis scenarios, in order to find out how to achieve the fundraising goal effectively through the text strategy. Upon the outbreak of a significant public crisis, the risk perception of the public fluctuates substantially due to uncertainty and information asymmetry, and individuals’ information searching and processing methods change accordingly (Lindell and Perry, 2012). Social media can be used by non-profit organizations to rapidly reach a large audience (Li et al., 2018) and provide information about their charitable crisis-relief projects. However, the factors that affect an individual’s decision to donate are complex and influenced by word of mouth of previous fundraisers, the experience of the executor, and the degree to which they are affected by the crisis events (Wan et al., 2017; Ki and Oh, 2018; Jin et al., 2016). However, once an individual has access to project information, the direct reference for their judgment is the project description. Previous research has highlighted the direct role that the textual information of project descriptions can play. For example, Majumdar and Bose (2018) found that the presence of rational and credible appeals in a message increases the likelihood of receiving a donation. And Some entrepreneurial research has proven that new entrepreneurs persuade private investors by manipulating the language (e.g., the tone and style) of business plans to increase their probability of being selected for further consideration or access to funds (Parhankangas and Ehrlich, 2014; Chan and Park, 2015). Therefore, in the context of a significant crisis event, where the public may not have access to additional trustworthy cues, we attempt to determine whether the quality of the project description can persuade the audience to donate through the forwarding times and whether the crisis involvement can moderate the framing effect of the textual information in the project description. Based on a large sample of data from Tencent, this research aims to further expand the research on text communication in the field of charitable crowdfunding.

## Theoretical Framework and Hypotheses Development

Fundraising is a persuasive activity that convinces potential donors to contribute to a worthy cause (Goering et al., 2011). Previous research attempted to understand why individuals donate from the perspectives of economics, psychology, sociology, marketing, and so on (Hibbert et al., 2007; Das et al., 2008; Majumdar and Bose, 2018). From the economic and psychological angle, people derive utility when they donate, and charity has been described as the consumption of “warm glow” (Andreoni, 1989) or the purchase of moral satisfaction (Kahneman and Knetsch, 1992). In the past, there are amount of research that focused on the donor’s demographic characteristics and their motivation to donate (Bretschneider et al., 2014). However, with the rise of Tencent and other crowdfunding platforms, such as GoFundMe, which are different from the traditional charity channels, the academic community began to pay attention to how persuasive text of charity request can improve the chance of receiving a donation. Take the Tencent platform as an example, strangers come forward to donate on the basis of the unknown seeker’s narration. Hence, the description of the project description (positive or negative framing, readability) is crucial.

**FIGURE 2:**
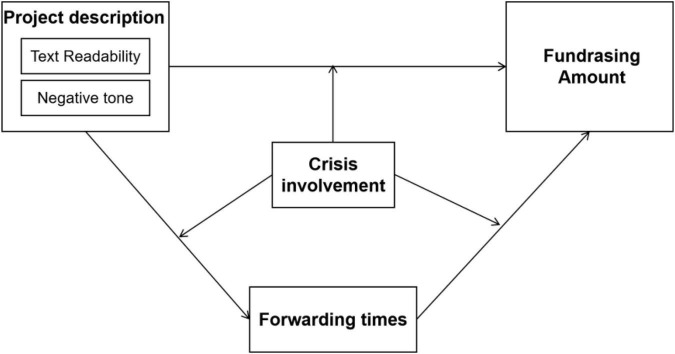
Conceptual framework.

We propose that non-profit organizations raise funds from online donors using persuasion through the project description. The research framework, shown in Figure 2, is constructed based on the cognitive theories of framing and information processing, to define the process of soliciting donations from supporters through the project description as a type of framing effect. Based on this framework, we explore whether textual information quality (i.e., the readability and the tone) is a prerequisite for fundraising amount by considering the mediating role of forwarding times and moderating role of crisis involvement.

### Readability and Fundraising Success

Readability serves as the cornerstone of text communication (Leong et al., 2002). This role has been discovered and validated in the field of financial accounting (e.g., Li, 2008; Loughran and McDonald, 2014; Asay et al., 2017) and marketing (e.g., Sawyer et al., 2008; Archak et al., 2011; Ludwig et al., 2013), finding that text readability can significantly affect the stock market value and product sales of listed firms.

We infer that the readability of a charitable project description affects fundraising success for the following two reasons. First, the readability of the project description can affect the donor’s reading time. Readable text improves the speed and ease in which the information receiver can browse and understand the meaning and purpose of the project (McKeown et al., 1992). Conversely, message receivers may not persevere with a poorly written project description. Tversky and Kahneman (1973) proposed that human information-processing capacity is limited in a specific environment; when individuals are faced with a large amount of information, they will selectively filter the information. Several scholars have begun to consider the phenomenon of social media information overload (e.g., Miritello et al., 2013; Bright et al., 2015). In the case of COVID-19, social media information about similar fundraising projects and the epidemic evolution is growing rapidly. As shown in Figure 1, since January 2020, COVID-19 has been a trending topic on networks and social media, and related charitable projects are launched continuously every day. Therefore, individuals’ tolerance for reading charitable project descriptions are limited and based on the premise that the project description can be easily understood.

Second, the readability of the project description reflects the qualifications of the fundraiser. Previous research on loan-based crowdfunding has examined the relationship between the readability of listed firms’ annual reports and their performance. Based on the Fog index of annual reports of listed firms, Li (2008) found that firms that provide more readable annual reports show stronger profitability. Furthermore, the obfuscation hypothesis proposes that underperforming firms will deliberately mask the true content of reports by reducing the readability of the text and by using unnecessarily complex vocabulary (Abu Bakar and Ameer, 2011). Similarly, research by Li (2008); Loughran and McDonald (2014), and Tan et al. (2015) on the annual reports of listed firms found that in the event of poor corporate performance, managers will increase the length of the annual report and add irrelevant information to confuse investors. Conversely, when the firm is performing well, annual reports provide simple explanations. We can, therefore, infer that the textual readability of a project description reflects the capabilities of the project sponsor. Tausczik and Pennebaker (2010) noted that the readability of text reflects the education, social status, and social class of information publishers. Therefore, initiators with rich experience in project execution and high credibility will use simple and understandable expressions. We posit that:

*Hypothesis 1: The better the readability of the charity project description, the greater the likelihood of fundraising success.*

### Negative Tone and Fundraising Success

Emotional framing has a significant influence on decision-making behavior, and its role in persuasion has long been a focus of researchers (e.g., Briñol et al., 2007; Sinclair et al., 2010). The emotional framework refers to the emotional implications of the arguments within the information presented. Scholars of narratology state that narration can affect human emotion and influence individuals to obtain and spread information (Rimmon-Kenan, 2003). This theory can be extended to the study of narrative text; that is, text can influence an individual’s cognition and emotional response to generate appeal while conveying information. Psychology scholars have also found the importance of tone to charity request (Smith and Petty, 1996; Marchand and Filiatrault, 2002; Hibbert et al., 2007; Das et al., 2008). Almost all requests are marked with a negative word highlighting the seeker’s distress, anxiety, or anger (Majumdar and Bose, 2018). Some of the negative appeals that have been studied in the charitable setting and that induce an empathetic reaction from the prospective donors include sadness, anger, fear, and guilt, and these serve as a motivational force to donate (Bagozzi and Moore, 1994; Marchand and Filiatrault, 2002; Vitaglione and Barnett, 2003; Hibbert et al., 2007). In fact, past research has shown that donations are higher when victims display sad facial expressions in charity advertisements (Small and Verrochi, 2009).

Research investigating the effects of persuasion from the perspective of individual emotional state and information emotional frame, states that an individual in an unhappy state is more likely to be persuaded by a negative tone of information. That is, an alignment between an individual’s emotional state and the emotional framework of the information can increase the persuasion effect (DeSteno et al., 2004). Therefore, as the tone of the project description can have a direct emotional impact on readers (Demers, 2011) and capture the general attitude that project owners use to describe their products or services (Larcker and Zakolyukina, 2012), we believe that a negative tone in the charitable project description in a crisis scenario has the potential to mobilize the empathy of individuals to donate. Hence, we posit that:

*Hypothesis 2: The more negative the project description, the greater the likelihood of charity fundraising success.*

### The Mediating Role of Forwarding Times

The public’s forwarding behavior on social media has become one of the important focuses of research in recent years. Forwarding can be seen as the establishment of a conversational process, with social media spreading the conversation across an open, interconnected network of participants, breaking down the confines of space and groups. The public charitable crowdfunding platform installed on social media also provides users with a forwarding function so that the fundraising information can be widely shared. Research on social media forwarding behavior focuses more on influencing factors, incentives of forwarding, forwarding content and other angles. For example, Suh et al. (2010) found that the more followers a blogger has, the easier it is to forward tweets posted by other users. In addition, the user’s social networks also play a very important role, as people are more inclined to retweet tweets from users they’ve ever forwarded (Himelboim and Golan, 2019). Boyd et al. (2010) pointed out that users usually retweet for some purpose, such as hoping to expand the scope of tweet dissemination, expressing their support and recognition of a certain point of view, and expecting to receive more attention, and they found that users will consider whether their fans are the target audience of the tweet content before deciding to retweet, aiming to enhance their own image. Botha and Reyneke (2013) found that users have preferences for forwarded content, and it is easy to forward content that is interesting or has emotional resonance. However, in the context of disaster, research on the role of public forwarding behavior on the chartable crowdfunding platform is relatively lacking. The information directly generated by the forwarding user is more real-time, which can more directly reflect the emotional changes and psychological conditions of the forwarder, and may have an impact on the emotions and cognition of other users in the process of dissemination, thereby promoting the fundraising of public welfare projects. Digging deeper into the role of such behaviors can help decision makers make the right judgments in emergency management. Hence, we posit that:

*Hypothesis 3: Forwarding times play the mediating role in the relationship between textual information (i.e., readability and negative tone) and fundraising amount.*

### The Moderating Role of Crisis Involvement

The moderating role of individual involvement is stressed in the literature on the process of persuasion (e.g., Coombs and Holladay, 2005; Choi and Lin, 2009; Claeys and Cauberghe, 2014). In the field of crisis communication, McDonald and Härtel (2000) defined the concept of perceived crisis involvement, which refers to the degree to which individuals comprehensively feel personal relevance. That is, the perception of the personal relevance of an issue determines the degree of an individual’s involvement with the issue. Scholars believe that involvement is related to situational and endogenous factors. Once the internal clues, such as knowledge and information, are extracted by individuals, involvement in a certain topic will be activated, and this level of involvement influences the amount and direction of their attention (Celsi and Olson, 1988), cognition, and information processing (McDonald and Härtel, 2000). Although most of the existing findings on crisis involvement are based on organizations, their theoretical basis and reasoning logic can provide us with an important reference to study the effect of individual involvement on information processing during a social crisis.

Similarly, we speculate that the degree of personal involvement in a social crisis affects the way individuals interpret information about a charitable project, which further affects the donation decision. According to the elaboration likelihood model, the degree of involvement will interact with specific information; individuals with high involvement will interpret the relevant information in depth, while an individual with low involvement will interpret the information formally. That is, crisis involvement can influence the value of crisis-related information (Claeys and Cauberghe, 2014). Moreover, crisis involvement can lead to greater attention to related information; for example, charitable projects for COVID-19 social assistance are more likely to attract the attention of those who are personally affected by the crisis. The information will have a greater impact on those personally affected due to their systematic information processing of the project description, thereby amplifying the effect of the project description on their donation behaviors. Hence, we posit that:

*Hypothesis 4: Crisis involvement plays a moderating role in the relationship between textual information (i.e., readability and negative tone) and fundraising amount.*

*Hypothesis 5: Crisis involvement furtherly moderates the mediating path of fundraising amount through the forwarding times.*

## Materials and Methods

### Sample and Data Collection

Tencent is the most influential social media tool in China, with 1.1 billion active users. As a platform that integrates and standardizes distributed public welfare information, Tencent charitable platform has great advantage. First, in WeChat’s payment function, “Tencent Philanthropy” is built-in, that is, users can participate in public welfare activities without going through the website or official account. Second, the same link for participating in public welfare can be obtained in different ways. Decentralizing the entrance to participating in public welfare can attract more people. Despite this, the influence of its official account should not be underestimated. Almost every time it publishes, there will be more than 100,000 readings, and the spread is extremely wide.

We collected all the charitable fundraising projects published on the platform between January 23 and February 29, 2020^2^. Each project has a fixed id number, and we checked the continuity of the id number to make sure we have collected all projects data. Then we manually read the project title and project description to find out all the projects about COVID-19. After eliminating three test projects and 45 projects without a clear fundraising target^3^, our final sample consisted of 205 charitable projects for COVID-19 relief launched on Tencent. By March 31, 2020, a total of 11,177,249 donors contributed CN¥1,575,096,774 to these projects, achieving 35.46% of the target amount of all projects. Based on the threshold value of the donation target, we determined the proportion of projects that achieved the project fundraising target. As shown in Table 1, even during a significant crisis that endangers each individual, few charitable projects achieved their goal. In addition, considering a charitable organization may use the same formats and patterns to write their descriptions for different projects: they may, for example, only replace a small part of the data and pictures in one description for one project to formulate a new one for another project. It follows that project descriptions from the same organization may not vary much in terms of readability and tone management. We counted the initiators of these 205 projects and found them from 198 charitable organizations, during which one institution initiated up to three fundraising projects, indicating that our sample is representative.

**TABLE 1:** Statistics of charitable fundraising projects.

Fundraising goal (10 thousand yuan)	Number of projects	Completion rate (%)
No clear goal	43	–
[1–10)	9	30.47
[10–50)	39	23.77
[50–100)	20	20.53
[100–500)	83	40.65
[500–1000)	27	64.78
[1000–3000]	25	66.80
11000	1	100.00
75000	1	0.02

A web crawler created in Python 8.0 was adopted to automatically capture project information, donor information, and media reports on the Internet and social media platforms (i.e., WeChat and Sina Weibo). Then, the readability (similarity and understandability) and keywords frequency were calculated, respectively, through Python’s language analysis module. COVID-19 statistics are from the official website of the National Health Commission of the People’s Republic of China.

### Measurement

#### Dependent Variable

The dependent variable is *fundraising amount* (FA), measured by the total donation amount of the project.

#### Independent Variables

##### Readability

Attention capacity in specific environments is limited (Tversky and Kahneman, 1973). According to the “7 ± 2 principle” proposed by Miller (1956), the human brain blocks complex information and can generally remember only 5–9 things in the short term. Complex text information produces information noise and increases the cost of information processing for the audience (Miller, 2010). Previous measurement of readability, such as the fog index (Fog Index), is represented by the average length of each sentence (ASL). What’s more, the punctuation mark can work to split the text (Shriberg et al., 2000) and the number of punctuation marks significantly affects the text content and sentence length (Roux, 2008). Therefore, we use the number of words in each pause^4^ to measure the complexity. The specific formula of complexity is as follows:


$${\text{Readability}}_{-\mathrm{complexity}}\,\left(\mathrm{RC}\right)=\frac{\mathrm{Number}\,\mathrm{of}\,\mathrm{words}}{\mathrm{Number}\,\mathrm{of}\,\mathrm{punctuation}\,\mathrm{marks}}$$

Second, we use the corpus word list, published by the Ministry of Education of the People’s Republic of China, as an integral part of understandability in text readability. The corpus word list contains 20 million Chinese characters, including 14,629 common words according to the number of occurrences. Following the research of Chen et al. (2018), understandability is measured by the proportion of the number of words included in the corpus word list in the project description. The specific formula of understandability is as follows:


$${\text{Readability}}_{-\text{understandability}}\,\left(\mathrm{RU}\right)=\frac{\mathrm{Number}\,\mathrm{of}\,\mathrm{common}\,\mathrm{words}}{\mathrm{Total}\,w\,\text{ords}}$$

##### Negative Tone

For each project description, the sentiment analysis extracts Chinese characters (excluding numbers, English characters, punctuations, URL, hashtags, and mentions), construct Chinese word segmentations, and obtain the sentiment scores of these word segments from the Chinese emotional vocabulary ontology. Chinese emotional vocabulary ontology (CEVO) is a Chinese ontology resource organized and labeled by the Information Retrieval Research Office of Dalian University of Technology. This resource describes a Chinese word or phrase from different perspectives, including word type, emotion type, emotion intensity and polarity. The emotion classification system of CEVO is built on the basis of Ekman. There are seven parts of word categories in the dictionary. These are Noun, Verb, Adjective, Adb, NW, IDIOM, and Prepositional Phrase. Each word corresponds to one of three polarities: neutral (5,376), positive (11,230), and negative (10,784). With the help of natural semantics processing tool, we identified the number of positive/negative words in project description based on CEVO. Then, we measure negative tone as the percentage the difference of negative and positive words in the project description. Specifically, it is calculated as follows:


$$\mathrm{Negative}\,\mathrm{tone}\,\left(\mathrm{NT}\right)\;=\frac{\mathrm{Number}\,\mathrm{of}\,\text{n}\,\mathrm{egative}\,\mathrm{words}-\mathrm{Number}\,\mathrm{of}\,\text{p}\,\mathrm{ositive}\,\mathrm{words}}{\mathrm{Total}\,\mathrm{words}}$$

#### Mediating Variable

**FIGURE 3:**
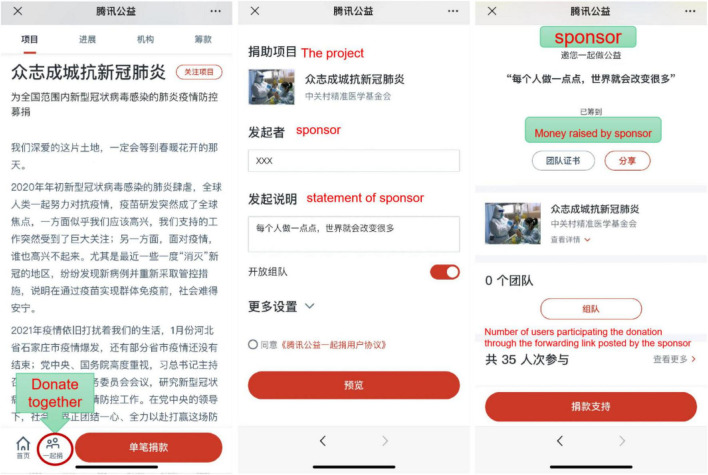
The function of “donate together”.

The mediating variable, *forwarding times* (FT), is measured by the number of donors who started “donate together.” As shown in Figure 3, “donate together” is a special function set up by Tencent public welfare platform. When the user decided to donate the project, they can choose whether to launch “donate together.” If the function was chosen, the user can spread this fundraising project to their social media friends, so as to expand the spread of project information.

#### Moderating Variable

Personal involvement/relevance pertains to how publics perceive importance of an object or their mental distance from a topic (Petty and Cacioppo, 1986; Jin et al., 2016). It can be found that the extent of involvement is closely related to the perceived crisis severity, and that the description of the severity of the epidemic needs to take into account comprehensively the number of confirmed cases as well as the total local population size. Hence, the moderating variable, *crisis involvement* (CI), is measured by the per cumulative number of confirmed COVID-19 cases from the previous day in the province where the donor target is located.


$$\mathrm{Crisis}\,\mathrm{involvement}\,\left(\mathrm{CI}\right)=\sum_{\text{i}=1}^{\text{n}}\frac{{\text{M}}_{\text{i}}}{l\,o\,c\,a\,l\,\_\,p\,o\,p\,u\,l\,a\,t\,i\,o\,n\,\_\,s\,i\,z\,e}/n$$

where M_I_ is the cumulative number of confirmed COVID-19 cases from the previous day in the province where the donor target *i* located and *n* is the total number of donors of a project.

#### Control Variables

We include variables that have been used in prior studies to control for individual prosocial behaviors (e.g., Hannah et al., 2011; Bolino and Grant, 2016; Gotowiec and van Mastrigt, 2019). The variables are *fundraising target, clear deadline, description length, images, plan length, initiator experience, executive expertise, recommended times*, *total confirmed cases nationwide, total recovered cases nationwide, total death cases nationwide, and media reports.*

The detailed measurements and sources of each variable are shown in Table 2.

**TABLE 2:** Measurement of variables.

Variable name	Measurement
1. Fundraising amount (FA)	The total fundraising amount of the project by March 31, 2020.
2. Readability	${\text{Readability}}_{-\mathrm{complexity}}\,\left(\mathrm{RC}\right)=\frac{\mathrm{Number}\,\mathrm{of}\,\mathrm{words}}{\mathrm{Number}\,\mathrm{of}\,\mathrm{punctuation}\,\mathrm{marks}}$
	${\text{Readability}}_{-\mathrm{understandability}}\,\left(\mathrm{RU}\right)=\frac{\mathrm{Number}\,\mathrm{of}\,\text{common}\,\mathrm{words}}{\mathrm{Total}\,\mathrm{words}}$
3. Negative tone	$\mathrm{Negative}\,\mathrm{tone}=\frac{\mathrm{Number}\,\mathrm{of}\,\text{n}\,\mathrm{egative}\,\mathrm{words}-\mathrm{Number}\,\mathrm{of}\,\text{p}\,\mathrm{ositive}\,\mathrm{words}}{\mathrm{Total}\,\mathrm{words}}$
4. Forwarding times	Log 10 (the number of donors who initiated “donate together”+1)
5. Crisis involvement	$\mathrm{Crisis}\,\mathrm{involvement}\,\left(\mathrm{CI}\right)=\sum_{\text{i}=1}^{\text{n}}\frac{{\text{M}}_{\text{i}}}{l\,o\,c\,a\,l\,\_\,p\,o\,p\,u\,l\,a\,t\,i\,o\,n\,\_\,s\,i\,z\,e}/n$
	Where M_i_ is the cumulative number of confirmed COVID-19 cases from the previous day in the province when the donor target *i* located; *n* is the total number of donors of a project.
6. Fundraising target	Log 10 (the amount initiator seeks to raise)
7. Clear deadline	1 = Deadline is marked on the page; 0 = Deadline is not marked on the page
8. Description length	Log10 (the number of words contained in project description)
9. Images	Log 10 (the number of images embedded in the project description+1)
10. Plan length	Log 10 (the number of words contained in project plan+1)
11. Initiator experience	Log 10 (the number of projects created by the initiator in the last year+1)
12. Executive expertise	Log 10 (the number of projects successfully executed by the executive in the last year+1)
13. Recommended times	The number of times it was set to “Recommend Items Today” by the platform
14. Other projects	Log 10 (the number of other projects initiated that day+1)
15. Total confirmed cases nationwide	log⁢ 10⁢(∑i=1nWin)
	Where *W*_*i*_ is the cumulative number of confirmed COVID-19 cases from the previous day nationwide when the donor *i* donated; *n* is the total number of donors of a project.
16. Total recovered cases nationwide	log⁢ 10⁢(∑i=1nRin)
	Where *R*_*i*_ is the cumulative number of recovered COVID-19 cases from the previous day nationwide when the donor *i* donated; *n* is the total number of donors of a project.
17. Total death cases nationwide	log⁢ 10⁢(∑i=1nDin)
	Where *D*_*i*_ is the cumulative number of death COVID-19 cases from the previous day nationwide when the donor *i* donated; *n* is the total number of donors of a project.
18. Media reports	log⁢ 10⁢(∑i=1nMediain)
	Where Media_i_ is the cumulative number of reports online (including the Web and Social media — Sina Blog and Weichat) from the previous day nationwide when the donor *i* donated; *n* is the total number of donors of a project.

### Model and Analysis

#### Mediation Effect Model

According to the analysis of the impact mechanism, the mediation effect model is introduced, and the impact of text information on the fundraising amount (FA) through the forwarding times (FT) is analyzed. In order to effectively eliminate heteroscedasticity, this paper uses the form of a double logarithmic function for estimation. The mediation effect model as follows:


(1) $${\text{lnFA}}_{\text{i}}=\alpha_{0}+\alpha_{11}\,\text{ln}\,R\,C_{i}+\alpha_{12}\,\text{ln}\,R\,U_{i}+\alpha_{13}\,\text{ln}\,N\,T_{i}+\alpha_{2}\,\text{ln}\,C\,V_{i}+\varepsilon_{1\,i}$$

(2) $${\text{lnFT}}_{\text{i}}=\beta_{0}+\beta_{11}\,\text{ln}\,R\,C_{i}+\beta_{12}\,\text{ln}\,R\,U_{i}+\beta_{13}\,\text{ln}\,N\,T_{i}+\beta_{2}\,\text{ln}\,C\,V_{i}+\varepsilon_{2\,i}$$

(3) $${\text{lnFA}}_{\text{i}}=\gamma_{0}+\beta_{11}\,\text{ln}\,R\,C_{i}+\gamma_{12}\,\text{ln}\,R\,U_{i}+\gamma_{13}\,\text{ln}\,N\,T_{i}+\gamma_{2}\,{\text{lnFT}}_{\text{i}}+\gamma_{3}\,\text{ln}\,C\,V_{i}+\varepsilon_{3\,i}$$

where *i* represents the charitable fundraising project; FA represents the amount raised; *RC* and *RU* represent two indicators of readability that is complexity and understandability, respectively; *NT* indicates the negative tone in the project description; *CV* are the control variables; ε represents the random error; _11,12,13_ indicate the total effect of complexity, understandability and negative tone on the amount of fundraising, respectively; _11,12,13_ indicate the direct effect of them, respectively; _11_×_2,12_×_2,13_×_2_ represent the mediating effect that represents text complexity, understandability, and negative tone transmitted through the forwarding times.

#### Moderated Mediation Model

In order to further test the regulatory effect of crisis involvement on the mediation effect, a regulated mediation effect estimation model is constructed as follows:


(4) $$\text{ln}\,F\,A_{i}=c_{0}+c_{11}\,\text{ln}\,R\,C_{i}+c_{12}\,\text{ln}\,R\,U_{i}+c_{13}\,\text{ln}\,N\,T_{i}+c_{2}\,\text{ln}\,C\,I_{i}+c_{31}\,\left(\text{ln}\,R\,C_{i}\times \text{ln}\,C\,I_{i}\right)+c_{32}\,\left(\text{ln}\,R\,U_{i}\times \text{ln}\,C\,I_{i}\right)+c_{33}\,\left(\text{ln}\,N\,T_{i}\times \text{ln}\,C\,I_{i}\right)+c_{4}\,\text{ln}\,C\,V_{i}+\varepsilon_{1\,i}$$

(5) $$\text{ln}\,F\,T_{i}=a_{0}+a_{11}\,\text{ln}\,R\,C_{i}+a_{12}\,\text{ln}\,R\,U_{i}+a_{13}\,\text{ln}\,N\,T_{i}+a_{2}\,\text{ln}\,C\,I_{i}+a_{31}\,\left(\text{ln}\,R\,C_{i}\times \text{ln}\,C\,I_{i}\right)+a_{32}\,\left(\text{ln}\,R\,U_{i}\times \text{ln}\,C\,I_{i}\right)+a_{33}\,\left(\text{ln}\,N\,T_{i}\times \text{ln}\,C\,I_{i}\right)+a_{4}\,\text{ln}\,C\,V_{i}+\varepsilon_{2\,i}$$

(6) ln⁢F⁢Ai=c0′+c11′⁢ln⁢R⁢Ci+c12′⁢ln⁢R⁢Ui+c13′⁢ln⁢N⁢Ti+c2′⁢ln⁢C⁢Ii+c31′⁢(ln⁢R⁢Ci×ln⁢C⁢Ii)+c32′⁢(ln⁢R⁢Ui×ln⁢C⁢Ii)+c33′⁢(ln⁢N⁢Ti×ln⁢C⁢Ii)+b1⁢ln⁢F⁢Ti+b2⁢(ln⁢F⁢Ti×ln⁢C⁢Ii)+b3⁢ln⁢C⁢Vi+ε3⁢i

where CI represents crisis involvement; the coefficients of (ln*RC*_*i*_×ln*I*), (ln*RU*_*i*_×ln*CI*_*i*_), (ln*NT*_*i*_×ln*CI*_*i*_), and (ln*FT*_*i*_×ln*CI*_*i*_) Ire used to measure the moderating effects; Under the moderating role of crisis involvement, the total effects of text complexity, understandability and negative tone on the fundraising amount are *c*_11_c_31_ln*CI*_*i*_, *I*c_32_ln*CI*_*i*_, and *I*_13_*c*_33_ln*CI*_*i*_; Under the moderating role of crisis involvement, the effects of complexity, understandability and negative tone on the forwarding times are *a*_11_*a*_31_ln*CI*_*i*_, *a*_12_*a*_32_ln*CI*_*i*_, and *a*_13_*a*_33_ln*CI*_*i*_, respectively; Under the moderating role of crisis involvement, the intermediary effects of the forwarding times on the amount of fundraising are (*a*_11_a_31_ln*CI*_*i*_)(*b*_1_*b*_2_*CI*_*i*_), (*a*_12_*a*_32_ln*CI*_*i*_)(*b*_1_*b*_2_*CI*_*i*_), (*a*_13_*a*_33_ln*CI*_*i*_)(*b*_1_*b*_2_*CI*_*i*_), respectively; c11′⁢c31′⁢ln⁢C⁢Ii, c12′⁢c32′⁢ln⁢C⁢Ii, c13′⁢c33′⁢ln⁢C⁢Ii indicate the direct effects of text complexity, understandability and negative tone on the amount of fundraising under the moderating role of crisis involvement.

## Results

We test our hypotheses through the sample of 205 COVID-19 charitable fundraising projects with a clear fundraising target initiated on Tencent from January 23, 2020, to February 29, 2020. Tables 3, 4 lists the results of the mediation effect model and that with regulation.

According to the Table 3, the model 3 indicates the coefficients of complexity, understandability and negative tone are −0.099, 0.068 and 0.402, respectively. It suggests that the textual information has the direct effect on the fundraising amount. The coefficient of complexity (β = 0.099,p < 0.001) is negative and significant, indicating that a complex project description decreases the total amount of charitable fundraising, while the coefficient of understandability (β = 0.068,p < 0.01) is positive and significant, suggesting that using common words to write the project description can increase the fundraising amount. That is, the better the readability of the project description (i.e., low complexity and high understandability), the greater the likelihood of raising fundraising, which supports Hypothesis 1. The coefficient of negative tone (β = 0.234,p < 0.001) is significant, suggesting Hypothesis 2 is supported. In model 2, coefficient for understandability (β = 0.221,p < 0.001) and negative tone (β = 0.407,p < 0.001) are positive and significant, and that for complexity (β = 0.098,p < 0.01) is negative and significant, indicating that forwarding times serves a significant mediating effect between textual information and fundraising amount and Hypothesis 3 is supported. Specifically, the indirect effects of complexity, understandability and negative tone on the amount of donations raised by the forwarding times were −0.039, 0.089, and 0.016, respectively, and the total effects were −0.138, 0.155, and 0.398, with mediating effects accounting for 28.2%, 57.4%, and 4.0%, respectively. Markowitz and Shulman (2021) found the predictive utility of word familiarity for online engagements and funding. Our results on complexity and understandability are consistent with the study. The results of the negative tone acting positively on fundraising amount differ from those found by Majumdar and Bose (2018). In their research on registered charities in online communities, they found that negative emotional appeal does not do the likelihood of receiving a donation. We believe that this may be due to differences study context. Our data were collected from January 23, 2020, to February 29, 2020 during the worst period of COVID-19 in China, The public would have more empathy with negative emotional appeal under a common viral threat.

**TABLE 3:** Estimation results of the mediation effect model.

Variables	Model 1 DV: FA	Model 2 DV: FT	Model 3 DV: FA
(Constant)	1.761***	1.627***	1.108***
RC	−0.138***	−0.098**	−0.099***
RU	0.155***	0.221***	0.066**
NT	0.398***	0.407***	0.234***
FT			0.402***
CV	Controlled	Controlled	Controlled
*R* ^2^	0.439	0.483	0.528
Adj *R*^2^	0.436	0.479	0.523
F-statistics	112.36	133.97	120.07

*p < 0.05, **p < 0.01, ***p < 0.001.

**FIGURE 4:**
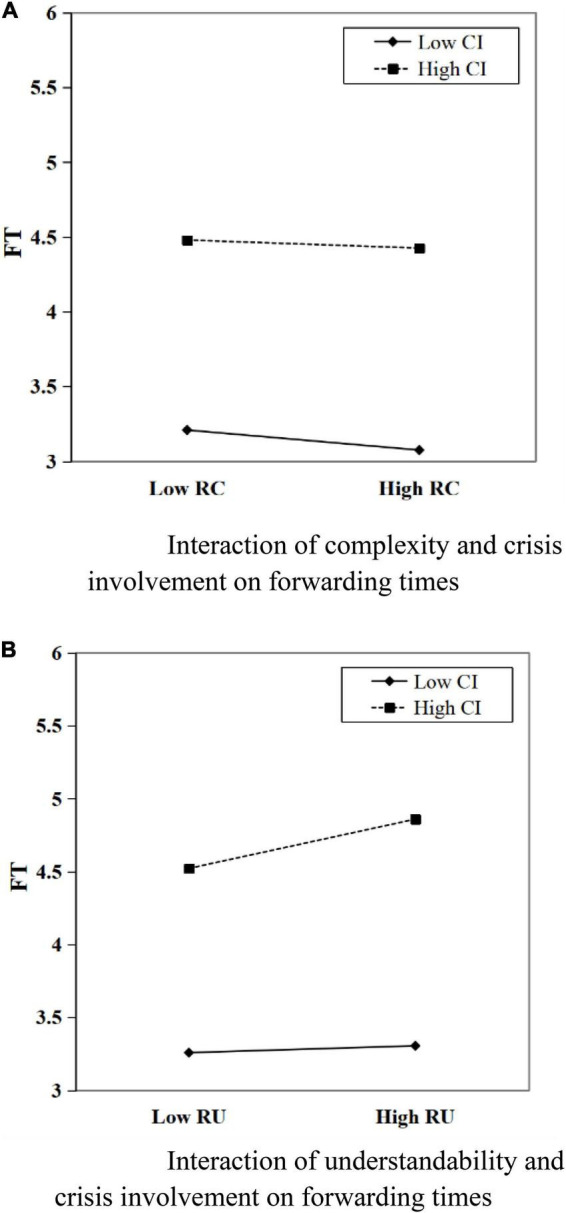
**(A)** Interaction of complexity and crisis involvement on forwarding times. **(B)** Interaction of understandability and crisis involvement on forwarding times.

**FIGURE 5:**
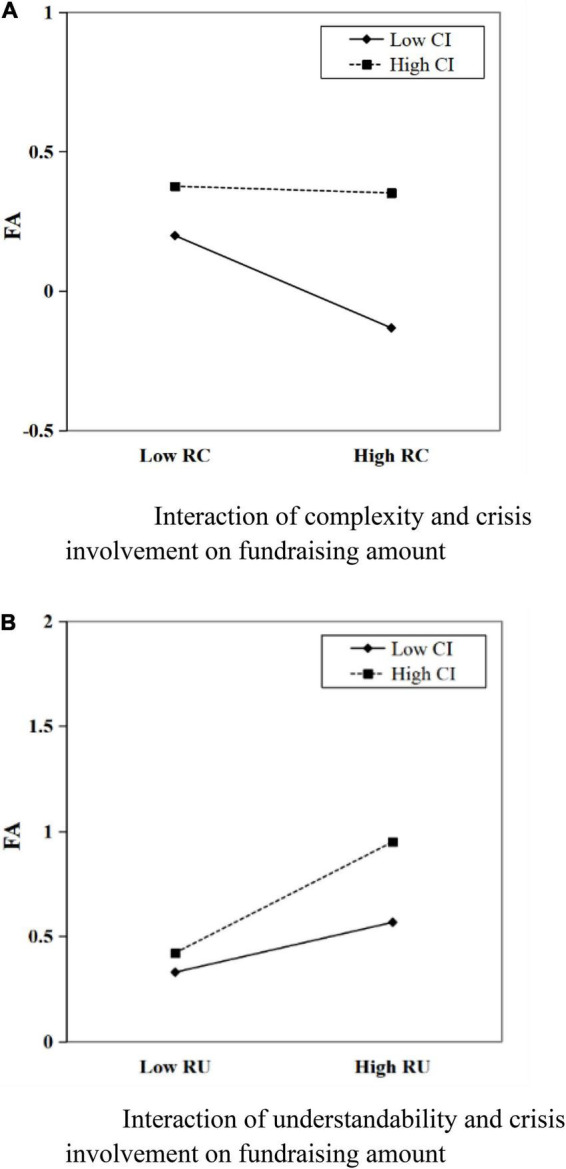
**(A)** Interaction of complexity and crisis involvement on fundraising amount. **(B)** Interaction of understandability and crisis involvement on fundraising amount.

Table 4 provides the estimation results of the mediating effect model with regulation. The regression results of model 6 show that the coefficient of the interaction term NTCI (i.e., *negativetone* = *crisisinvolvement*) is not significant, and the regression coefficient of the interaction term RCCI (i.e., complexity = *crisisinvolvement*) and RUCI (i.e., understandability = *crisisinvolvement*) is significantly positive. It shows that the degree of crisis involvement moderates’ complexity and understandability to the direct path of the amount of fundraising, suggesting that Hypothesis 4 is partially verified. The interaction plot in Figure 4A reports a positive interaction between complexity and crisis involvement (β = 0.046,p < 0.05) on fundraising amount. It indicates that the negative effect of the complexity of the project description can be weakened when the crisis involvement is high. Figure 4B indicates a positive interaction between understandability and crisis involvement (β = 0.050,p < 0.001) on fundraising amount. It shows that when the crisis involvement is in a high degree, the positive effect of understandability on the amount of donations raised will be further amplified. Further, according to the significance of the interaction term RCCI and RUCI of model 5 and the coefficients of the interaction terms of model 6, it can be seen that the degree of crisis involvement affects the indirect path of the amount of fundraising through the forwarding times, and the moderating effect is significant, in line with hypothesis H5. Specifically, the interaction plots of Figures 5A,B report the positive interactions between readability (i.e., complexity and understandability, respectively) and crisis involvement. It is explained that the degree of crisis involvement can reduce the negative effect of text complexity on the forwarding times, and thus reduce the negative effect on the amount of fundraising. In addition, crisis involvement amplifies its positive effect on the forwarding times by amplifying the positive effect of understandability on the forwarding times, which in turn amplifies its positive effect on the amount of donations raised.

**TABLE 4:** Estimated results of the moderated mediation effect model.

Variables	Model 4 DV: FA	Model 5 DV: FT	Model 6 DV: FA
(Constant)	1.531***	3.318***	0.316***
RC	−0.153***	−0.058***	−0.141**
RU	0.125***	0.029***	0.119***
NT	0.601***	0.188***	0.562***
CI	0.111***	0.417***	0.024***
FT			0.208***
RCCI	0.046***	0.012**	0.046**
RUCI	0.051**	0.046**	0.050***
NTCI	–0.070	0.053	–0.097
FTCI			0.019***
CV	Controlled	Controlled	Controlled
*R* ^2^	0.456	0.507	0.544
Adj *R*^2^	0.447	0.499	0.534
F-statistics	51.07	62.77	56.31

*p < 0.05, **p < 0.01, ***p < 0.001.

## Discussion and Conclusion

The success of charitable crowdfunding warrants research, especially during a public crisis, as it can serve as an efficient tool for non-profit organizations to raise resources and participate in crisis relief. By using a large dataset obtained from Tencent after the outbreak of COVID-19, we examine the influence of the project description on fundraising amount. We view the process by which non-profit organizations raise funds from online donors as a persuasion process through project descriptions and identify two kinds of exemplary antecedents from project description: text readability and negative tone, which serve as the information framing effect and the emotional framing effect, respectively. We then investigate the mediation role of forwarding times, as affective response to the text might explain forwarding times, which in turn affects money raised by increasing the visibility of the campaign. On this basis, the moderating role of one’s crisis involvement is tested during this process, as individuals with a high degree of crisis involvement will systematically process the information and make in-depth interpretations. The empirical results indicate that the complexity of the description will reduce the fundraising amount, while understandability and negative tone help to improve the success. Furthermore, we found that forwarding times played an important mediating role in this process. Then the buffer effect of crisis involvement on the negative effect of complexity was validated, and its amplified effect on the positive effects of understandability was also verified.

This study contributes to the crowdfunding literature in several ways. First, textual information is becoming increasingly important in the field of crowdfunding (Parhankangas and Renko, 2017). By exploring charitable projects with a focus on the information content of project descriptions in the scenario of a public crisis, we expand the research scenarios of such issues and combine scenario features, such as crisis involvement, to provide new perspectives for such future studies.

Second, most studies on the readability of the text focused on the English language. Due to differences in expression in different languages, the methods used to measure the readability of English text cannot be directly applied to Chinese text analysis (Hu et al., 2017). In the current research, we constructed a text readability index that can extract text information from a large number of Chinese natural languages and provide a new insight for text analysis in the Chinese language context.

Third, we expanded the research scenario of persuasion theory by studying the roles of information and emotional framing effects on the crowdfunding amount. How to effectively persuade the public to make donations is very important (Hibbert et al., 2007). The results highlight the importance of project descriptions and provide insights for non-profit organizations to focus on information and emotional framing of project descriptions when they launch charitable projects on crowdfunding platforms. Moreover, this can be applied to further crowdfunding research to enhance predictive capabilities.

Finally, the moderating effect of donors’ crisis involvement on fundraising success is noteworthy. We verified that high crisis involvement will stimulate the systemic information processing of individuals, thereby the framing effects of the project description are amplified. This finding extends the research on individuals’ issue involvement. There is currently no theoretical framework that uses individuals’ crisis involvement as a moderating variable that regulates the persuasive outcome of information and emotional frames. Therefore, the results of this study indicate the potential use of this variable in current and future theoretical frameworks.

Our study also has practical implications for the effective combination of information and emotional framing effects for individuals of different involvement levels. It provides an important reference for non-profit organizations to better frame information within the project description, to improve the participation of information receivers when seeking donations through online crowdfunding platforms. According to previous research on information framing, the information that can be verified by donors takes up a large weight, such as the experience and ability of initiators and executors, and it is costly for information receivers to verify the credibility of this information. The findings of this study prove that the text can reflect the capabilities of initiators, which is an effective and low-cost supplement to the traditional information framing model. Therefore, non-profit organizations should not only emphasize their credible qualifications and execution experience but also consider the readability and tone of the description, which will significantly improve the success of fundraising. Our research also reminds social media users how to make better decisions based on the textual quality of project descriptions when they are exposed to information about relevant charitable projects. Our results provide meaningful insights to researchers, project initiators (especially non-profit organizations), and online donors to better understand the importance of project descriptions and their influence on funding success during a crisis scenario.

Some limitations of this study offer suggestions for future research. First, we conduct our studies base on a single online crowdfunding platform. However, the charitable crowdfunding market is growing, and several crowdfunding platforms are not bound to social media for disseminating project information. This may limit the generalizability of our findings. Future research may test the framework and results of this study by using other charitable crowdfunding platforms bound to social media, such as Sina Weibo, and conduct comparative research between platforms that are bound to social media and those that are not. Even more, the qualitative analysis through a combined methodology, through meaning analysis, could be interesting in future studies. Furthermore, we suggest comparing the weight differences between the role of project description and the role of social media dissemination breadth. Second, we studied the moderating effect of individual crisis involvement. However, several other factors affect the information processing of individuals in a crisis, such as the attribution of responsibility for the crisis, the emotional state of individuals, and the physical and social connections with the affected areas. Future research may extend the textual analysis to individuals’ updates on social media about the crisis, to explore other influencing factors that may be different from traditional research and enrich relevant research on risk communication.

## Data Availability Statement

The raw data supporting the conclusions of this article will be made available by the authors, without undue reservation.

## Author Contributions

LL and WJ contributed to the material preparation, data collection, and analysis. JX wrote the first draft of the manuscript. All authors contributed to the study conception and design, commented on previous versions of the manuscript, and read and approved the final manuscript.

## Conflict of Interest

The authors declare that the research was conducted in the absence of any commercial or financial relationships that could be construed as a potential conflict of interest.

## Publisher’s Note

All claims expressed in this article are solely those of the authors and do not necessarily represent those of their affiliated organizations, or those of the publisher, the editors and the reviewers. Any product that may be evaluated in this article, or claim that may be made by its manufacturer, is not guaranteed or endorsed by the publisher.

## Footnotes

1
http://gongyi.qq.com

2 The first project on COVID-19 assistance launched on Tencent was on January 23, 2020. Hence, we chose January 23, 2020 as the starting time of our sample.

3 The main reason for excluding the projects with no clear goal from the sample was to remove the bias from charities that are less likely to succeed or have a different focus. For example, many of the excluded charity projects are part of the guardian of the angels of war, which was launched in late February and aims to help health workers in the aftermath of the crisis.

4 The paused punctuation marks include the full stop, question mark, exclamation mark, comma, colon, and semicolon.
